# SARS-CoV-2 infection induces robust germinal center CD4 T follicular helper cell responses in rhesus macaques

**DOI:** 10.21203/rs.3.rs-51545/v1

**Published:** 2020-08-14

**Authors:** Sonny Elizaldi, Yashavanth Shaan Lakshmanappa, Jamin Roh, Brian Schmidt, Timothy Carroll, Kourtney Weaver, Justin Smith, Jesse Deere, Joseph Dutra, Mars Stone, Sergej Franz, Rebecca Sammak, Katherine Olstad, J. Rachel Reader, Zhong-Min Ma, Nancy Nguyen, Jennifer Watanabe, Jodie Usachenko, Ramya Immareddy, JoAnn Yee, Daniela Weiskopf, Alessandro Sette, Dennis Hartigan-O’Connor, Stephen McSorley, John Morrison, Nam Tran, Graham Simmons, Michael Busch, Pamela Kozlowsk, Koen van Rompay, Christopher Miller, Smita Iyer

**Affiliations:** University of California, Davis; University of California, Davis; University of California, Davis; University of California, Davis; University of California, Davis; Louisiana State University Health Sciences Center; Louisiana State University Health Sciences Center; University of California, Davis; University of California, Davis; Vitalant Research Institute; University of California, Davis; University of California, Davis; University of California, Davis; University of California; University of California, Davis; University of California, Davis; University of California; University of California, Davis; University of California, Davis; University of California; Division of Vaccine Discovery, La Jolla Institute for Allergy and Immunology, La Jolla, CA.; La Jolla Institute for Immunology; University of California, Davis; UC Davis; University of California; University of California, Davis; Vitalant Research Institute; University of California, San Francisco; Louisiana State University Health Sciences Center New Orleans; UC Davis; University of California, Davis; University of California Davis School of Veterinary Medicine and California National Primate Research Center.

**Keywords:** SARS-CoV-2, Adaptive Immunity, Innate Immunity, SARS-CoV-2 specific antibody, SARS-CoV-2 specific T cells, Convalescent plasma, Nucleocapsid, Spike

## Abstract

CD4 T follicular helper (T_fh_) cells are important for the generation of durable and specific humoral protection against viral infections. The degree to which SARS-CoV-2 infection generates T_fh_ cells and stimulates the germinal center response is an important question as we investigate vaccine options for the current pandemic. Here we report that SARS-CoV-2 infection resulted in transient accumulation of pro-inflammatory monocytes and proliferating T_fh_ cells with a T_h_1 profile in peripheral blood. CD4 helper cell responses were skewed predominantly toward a T_h_1 response in blood, lung, and lymph nodes. We observed the generation of germinal center T_fh_ cells specific for the SARS-CoV-2 spike (S) and nucleocapsid (N) proteins, and a corresponding early appearance of antiviral serum IgG antibodies. Our data suggest that a vaccine promoting T_h_1-type T_fh_ responses that target the S protein may lead to protective immunity.

## Introduction

As of July 30th, 2020, SARS-CoV-2 has resulted in more than 17 million infections and more than half a million deaths, globally^1^. Unanticipated post-infection complications, such as multisystem inflammatory syndrome pose a serious threat^2^. An effective vaccine is paramount, and several SARS-CoV-2 vaccine candidates are in various phases of human testing worldwide^3–5^. The most effective vaccines induce antibodies that provide long-term protection^6^ and vaccines using attenuated virus elicit the most persistent antibody responses; therefore, understanding the immunological mechanisms characteristic of controlled SARS-CoV-2 infection is foundational to the selection of a vaccine capable of abating the pandemic^7,8^.

Generation of persistent immunity hinges on CD4 T follicular helper cells (T_fh_). We and others have demonstrated that peripheral CD4 T_fh_ cells predict antibody durability in the context of HIV and influenza vaccines^9–11^. While studies in humans demonstrate induction of T_fh_ cells in COVID-19 patients^12–14^, the impact of SARS-CoV-2 infection on the generation of germinal center T_fh_ cells is currently unknown. This is a detrimental gap in knowledge as understanding early correlates of durable antibodies, specifically those that circulate in peripheral blood, will aid in the ultimate selection of effective vaccine candidates. SARS-CoV-2-specific CD4 T cells responding to spike proteins have been observed in the peripheral blood of recovered patients^15,16^. Similar observations have been made with the 2002 SARS-CoV virus^17,18^, and studies in mouse models have demonstrated a critical role for CD4 T cells in viral clearance^19^. Together, these data emphasize the need to understand CD4 T_fh_ responses following SARS-CoV-2 infection.

Because healthy rhesus macaques infected with SARS-CoV-2 resist immediate re-challenge with the virus^20–22^, we hypothesized that understanding the CD4 T_fh_ and germinal center (GC) response following exposure to SARS-CoV-2 will provide a framework for understanding immune mechanisms of protection. Here we report that, following infection with SARS-CoV-2, adult rhesus macaques exhibited transient accumulation of activated, proliferating T_fh_ cells with a T_h_1 profile in their peripheral blood. Perhaps more pertinent to SARS-CoV-2 as a respiratory virus, infection elicited robust GCs with SARS-CoV-2- reactive T_fh_ cells within the mediastinal lymph nodes. The comprehensive immune analysis in a controlled animal model of mild diseases adds to our understanding of immune responses to SARS-CoV-2; the data suggest that vaccine platforms inducing T_h_1-T_fh_ responses are likely to succeed in eliciting durable humoral responses.

## Results

### Experimental Design

**Figure 1:**
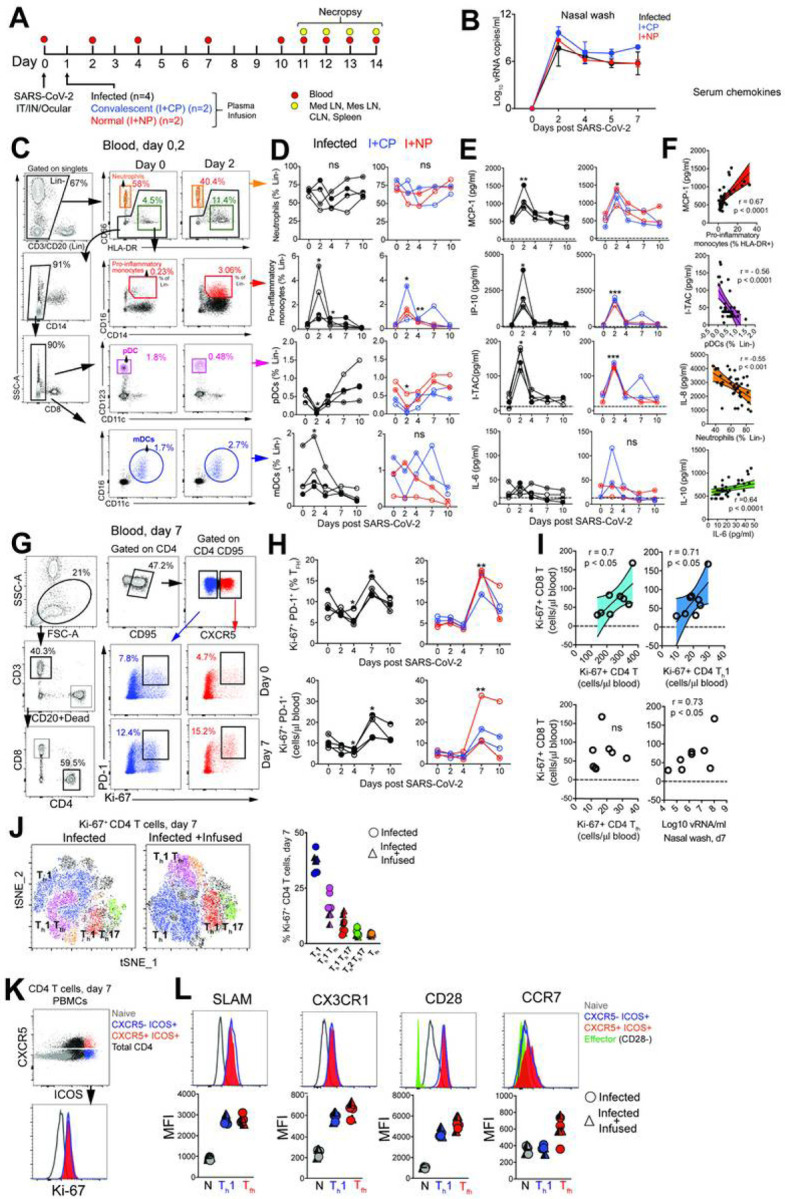
SARS-CoV-2 infection leads to a rapid and transient shift in innate immune responses and increases the number CD4 T follicular helper cells in peripheral blood. (A) Rhesus macaques inoculated with SARS-CoV-2 were infused with COVID-19 convalescent plasma (I+CP), or normal plasma (I+NP) or did not receive plasma (infected). (B) Mean viral RNA (+range) in nasal washes (C) Gating strategy for innate immune subsets in blood. (D) Kinetics of innate immune responses (*p< 0.05, **p< 0.01 relative to Day 0) (E) Serum chemokines MCP-1, IP-10, and I-TAC (*p< 0.05, **p< 0.01, ***p< 0.001 relative to Day 0). (F) Correlation of innate immune cells against chemokines, and IL-10 vs IL-6. (G) Gating strategy to capture Ki-67+ PD-1+ CD4 T cells in blood. (H) Kinetics show frequency and absolute counts of Ki-67+ PD-1+ CD4 Tfh cells (*p< 0.05, **p< 0.01 relative to Day 0) (I) correlation plots of Ki-67+CD8 T cells against Ki-67+ CD4 subsets, and vRNA (all day 7) (J) tSNE plot of CD4 Ki-67+ events at Day 7 from infected (16,197 events) and infected + infused animals (22,406 events); dot plot shows frequency of Ki-67+ CD4 T cell subsets. (K-L) Histograms and median fluorescence intensity (MFI) dot plots illustrate relative expression of SLAM, CX3CR1, CD28, and CCR7 within four different populations identified at Day 7. Unique symbols identify animals in each of the experimental groups.

To achieve our primary objective of assessing whether SARS-CoV-2 elicits T_fh_ cells and germinal-center responses, we challenged eight adult rhesus macaques (4–5y, Table S1) with a high-dose of SARS-CoV-2 (2 × 10^6^ PFU). Virus was administered via the intranasal, intratracheal, and ocular routes. Infection was monitored by following the quantity of viral RNA (vRNA) in nasal washes using qRT-PCR. Of the eight infected animals: four did not receive plasma infusion (Infected), two animals were infused with COVID-19 convalescent human plasma 24 hours following inoculation (I + CP), and two animals were infused with an identical volume of normal plasma lacking antibodies to SARS-CoV-2 (I + NP). (Fig. 1A). Pooled CP demonstrated a neutralizing titer of 1:1,149; but due to the estimated 50-fold dilution after infusion (based on 4 ml/kg infusion volume and ~ 20% extracellular fluid), neutralizing activity in macaque sera 24 hours post infusion fell below the limit of detection (1:40) of the neutralization assay. Consistently, CP administration did not blunt acute viral loads and high vRNA levels were observed in all animals (Fig. 1B). Histopathological lesions of the lungs confirmed multifocal to locally extensive interstitial pneumonia of mild to moderate severity in infected animals (Figure S1A). However, these histological changes were not accompanied by fever, weight loss, or any other signs of clinical disease (Figure S1B). None of the animals developed acute respiratory distress. In summary, infection of healthy adult rhesus macaques with SARS-CoV-2 resulted in high viremia but generally produced no overt signs of clinical illness, providing a framework to investigate development of protective immune responses.

#### SARS-CoV-2 infection leads to a rapid and transient shift in innate immune responses and increases the number of CD4 T follicular helper cells in peripheral blood.

Evaluation of innate immune cell subsets in the peripheral blood (Fig. 1C) revealed no significant changes in either the proportion or absolute counts of neutrophils over time (Fig. 1D). However, rapid and divergent changes in specific myeloid cell subsets were observed. While CD14 + CD16 + pro-inflammatory monocytes significantly increased at Day 2 with a corresponding increase in CX3CR1 expression (Figure S1C), pDCs decreased in peripheral blood. We also noted a significant increase in myeloid DCs (mDC) within the infected group. Pro-inflammatory chemokines monocyte chemoattractant protein (MCP-1), interferon Y-induced protein-10 (IP-10), interferon inducible T cell a chemoattractant (I-TAC) were significantly elevated at Day 2 and returned to baseline levels soon thereafter (Fig. 1E). We did not observe significant elevations in pro-inflammatory cytokines interleukin (IL)-6 (Fig. 1E). We observed a direct relationship between serum MCP-1 levels and pro-inflammatory monocytes over the course of infection, while pDCs and neutrophil frequencies were inversely related to I-TAC and IL-8 levels respectively (Fig. 1F). While no statistically significant changes occurred with IL-6 or IL-10, both cytokines were correlated over the course of infection. The brisk and transient innate immune dynamics following exposure to SARS-CoV-2 are consistent with the minimal changes observed in body weight and oxygen saturation levels and mild overall disease pathology.

To assess the increase in CD4 T_fh_ cells attributable to SARS-CoV-2, we profiled peripheral blood to capture effector T cell responses. No evidence of general lymphopenia was observed (Figure S1D). Frequency and absolute counts of activated CXCR5^+^ CD4 T_fh_ cells, identified by co-expression of Ki-67 and PD-1, significantly increased in all animals at Day 7 regardless of plasma intervention (Fig. 1G–H). At the apex of the effector response, Ki-67^+^ CD4 T cells, specifically the T_h_1 but not the T_fh_ subset was strongly associated with proliferating CD8 T cells (Fig. 1I). In turn, we observed strong antigen-dependent induction of CD8 T cells evidenced by the association between SARS-CoV-2 vRNA from nasal washes and proliferating (Ki67+) CD8 T cells.

Evaluation of infection-induced changes in CD4 T cell differentiation at Day 7 revealed a strong phenotypic shift to T_h_1 effectors (CXCR3+), T_h_1 polarized T_fh_ cells (CXCR3 + CXCR5+) and T_h_1 T_h_17 (CXCR3 + CCR6+) CD4 T cells (Fig. 1J, Figure S2A). Correspondingly, the data showed accumulation of CD4 T_h_1 cells at Day 7 (Figure S2B). While T_h_2 CD4 cells did not peak at Day 7, there was an increase of T_h_17 CD4 cells (Figure S2C–D). Using the acute activation marker, inducible costimulator (ICOS), to identify proliferating (Ki67+) CD4 T cells at Day 7 (Fig. 1K), we found that ICOS + CXCR5- and CXCR5 + CD4 T cells subsets expressed the T_h_1 marker signaling lymphocyte adhesion molecule (SLAM) and the effector molecule CX3CR1, a marker potentially for newly generated memory CD4 T cell subsets, consistent with their activation status (Fig. 1L). Neither the ICOS + CXCR5- nor the CXCR5 + CD4 T cell subsets downregulated CD28 and both subsets expressed CCR7 at levels comparable to or greater than naive cells, indicative of a lymph node-homing phenotype. To assess CD4 T cell functionality, cytokine production was evaluated *ex vivo* following stimulation with PMA and ionomycin. Two distinct CD4 T cells were identified - a degranulating CD107a + b subset with the majority of degranulating CD4 T cells expressing interferon gamma (IFNy) and TNFa but not IL-2 or IL-17; and an IL-21-producing subset (Figure S2E). In contrast, the majority of IL-21-expressing cells produced IL-2, IL-17 and co-produced TNFa and IFNy. Thus, CD4 T cell polyfunctionality was preserved during SARS-CoV-2 infection. Despite the increase in activated T_fh_ cells, levels of CXCL13 did not increase significantly following SARS-CoV-2 infection (Figure S2F).

#### CD4 T _fh_ cells targeting the spike (S) and nucleocapsid (N) proteins are generated following SARS-CoV-2 infection.

**Figure 2:**
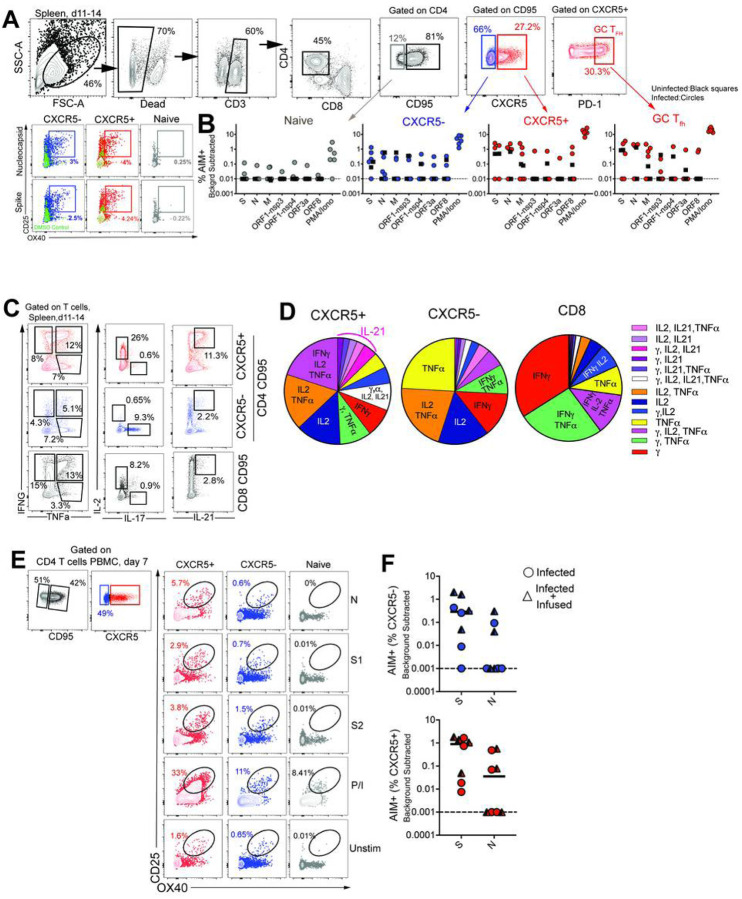
CD4 Tfh cells targeting the spike (S) and nucleocapsid (N) are generated following SARS-CoV-2 infection (A) Gating to identify SARS-CoV-2 specific CD4 T cells following stimulation with peptide megapools (B) Scatter plot showing AIM+ CD4 subsets. Dashed line represents undetectable responses assigned a value of 0.01% (C) Cytokine profiles (IFNy, IL-2, TNFa, IL-17, IL-21) of CXCR5+, CXCR5-, and CD8+CD95+ T cells) in spleen following P/I stimulation. (D) Pie chart shows T cell polyfunctionality. (E) Gating to identify SARS-CoV-2 specific CD4 T cells in PBMCs. (F) AIM+ CXCR5- and CXCR5+ CD4 subsets in PBMCs at Day 7. Black squares denote SARS-CoV-2 unexposed animals.

Based on the significant increase in systemic CD4 T_fh_ cells following SARS-CoV-2 exposure, we sought to understand splenic involvement during the germinal center phase of the immune response. To this end, we quantified GC T_fh_ cells in the spleen at necropsy and compared the values to those seen in animals that had not been exposed to SARS-CoV-2. The results suggested the initiation of a GC response within the spleen following infection (Figure S3A). We observed that the majority of the GC T_fh_ cells did not express Foxp3 indicating that GC T_fh_ cells predominated over the GC T follicular regulatory cell (T_fr_; CXCR5+, PD-1++, Foxp3+) subset (Figure S3B, Tregs were defined as CD95 + CXCR5- Foxp3+). To conclusively assess SARS-CoV-2-induced responses, we stimulated cryopreserved splenocytes with mega pools - overlapping peptides covering multiple T cell epitopes in S, N, and membrane (M) proteins, and spanning the open reading frames (ORF1,3,8) of SARS-CoV-2. PMA/Ionomycin was used as a positive control while DMSO-treated cells served as negative controls. Using activation induced marker (AIM) assay, SARS-CoV-2-specific CD4 T cells were identified based on co-expression of 0X40 and CD25 (Fig. 2A, Table S2).

Following subtraction of AIM + responses in DMSO-treated cells, CD4 T cell responses to S and N were detected. Furthermore, PD-1 + + GC T_fh_ cells, reactive to S, N, and M were observed indicative of SARS-CoV-2-induced GC response in the spleen (Fig. 2B). It should be noted, however, that responses to S, N, M were also detected in unexposed animals suggestive of cross-reactive T cells to endemic coronaviruses, as has been reported in humans^15,23^. Evaluation of CD4 T cell polyfunctionality in the spleen by ICS in response to PMA/Ionomycin stimulation revealed that CXCR5 + CD4 T cells were clearly distinguishable from CXCR5- subsets in their ability to co-produce IFNy, IL-2, TNFa, and IL-21. In contrast, the CXCR5-subset produced little IL-21 yet was able to co-produce IL-2 and TNFa, or, alternatively, either IFNy, IL-2, or TNFa. In contrast, CD8 T cells were predominantly IFNy producers (Fig. 2C–D). Consistent with data from the spleen, antigen-specific responses against S and N were also observed in peripheral blood at Day 7 (Fig. 2E–F). Together, these data demonstrate that S- and N-specific CD4 T_fh_ cells are elicited following SARS-CoV-2 infection.

#### SARS-CoV-2 infection induces germinal center responses in mediastinal lymph nodes.

**Figure 3:**
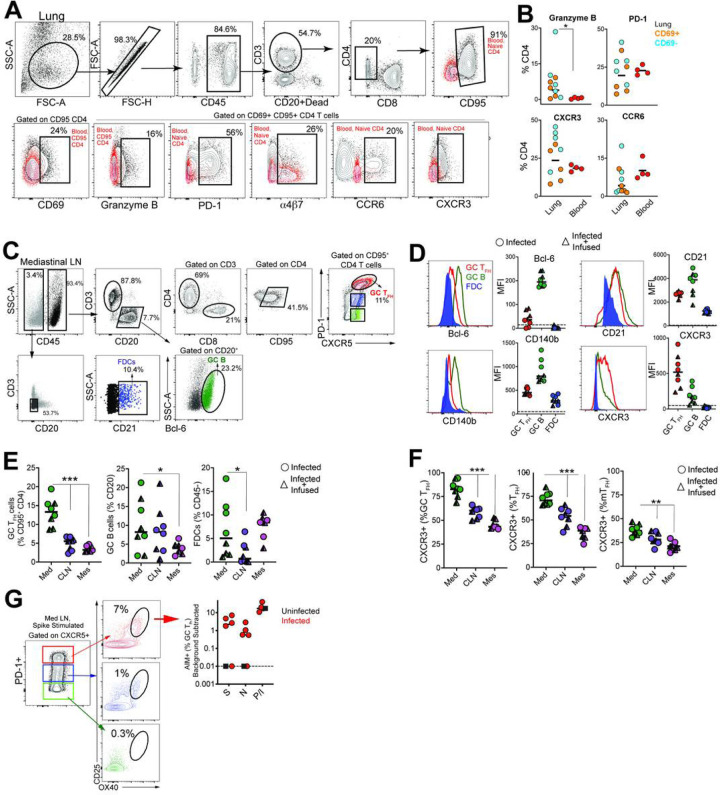
SARS-CoV-2 infection induces germinal center responses in mediastinal lymph nodes. (A) Gating to identify CD4 T cells in lung (B) Dot plots show Granzyme B, PD-1, CXCR3, CCR6 expression on CD69- and CD69+ subsets in lung and CD95+ CD4 T cells in blood (n=5). (C) Gating for GC Tfh cells, GC B cells, and FDCs. (D) Expression of Bcl-6, CD21, CD140b, and CXCR3. (E) Frequency of GC Tfh cells, GC B cells, FDCs significantly higher in mediastinal lymph node (*p< 0.05, ***p< 0.001). (F) Majority of GC Tfh cells in mediastinal lymph nodes express CXCR3 (**p< 001; ***p< 0.001). (G) Flow plot of PD-1+ CXCR5+ (GC) Tfh cells shows AIM+ cells following stimulation with spike; scatter plot shows specificity of GC Tfh cells to SARS-CoV-2 spike and nucleocapsid. The dashed line represents undetectable responses assigned a value of 0.01 %. Black squares denote SARS-CoV-2 unexposed animals.

Having established that SARS-CoV-2 stimulates the production of CD4 T_fh_ cells, we next assessed whether T_h_1 effector CD4 T cells were induced in the lung. Subsequent to collagenase digestion, single-cell suspensions isolated from the lung were stained with a panel of markers to delineate activated CD4 T cells. We evaluated expression of Granzyme B and PD-1, both antigen-induced activation markers; mucosal homing receptors a_4_ß_7_, CCR6, and the T_h_1 receptor CXCR3 within CD69 + and CD69- CD4 T cell subsets (Fig. 3A). The expression pattern of Granzyme B, PD-1, and CXCR3 in lung CD4 T cells was indicative of a T_h_1 effector CD4 response (Fig. 3B). Furthermore, histopathology of the lung showed development of Bronchus-associated lymphoid tissue (Figure S4A) substantiating induction of adaptive immune responses. Gross examination of the mediastinal lymph nodes was consistent with lymphadenopathy (Figure S4B). Single cell suspensions obtained from lymph nodes were stained with a panel of markers to define GC T_fh_ cells, GC B cells, and follicular dendritic cells (FDCs). As illustrated, mediastinal lymph nodes showed a distinct CXCR5 + PD-1 + + GC T_fh_ subset and Bcl-6 + CD20 + GC B cells (Fig. 3C). FDCs were identified based on expression of the complement receptor CD21 (clone B-Ly4; Figure S4C), within the CD45- CD3- CD20- cell population^24^. The number of FDCs strongly correlated with the frequencies of both GC B cells and GC T_fh_ cells (Figure S4D).

Quantifying the expression of canonical GC markers showed that Bcl-6 was exclusively expressed by GC B cells and to a lesser extent by GC T_fh_ cells (Fig. 3D). FDC markers CD21 and platelet-derived growth factor receptor b (CD140b)^25^ were also expressed by GC T_fh_ and B cells (Fig. 3D). An increase in expression of the T_h_1-chemokine receptor, CXCR3, on GC T_fh_ cells was consistent with the phenotype of cells responding to viral infection. While GC B cells displayed heterogeneity in CXCR3 expression, FDCs were uniformly negative for this marker. The increased number of GC T_fh_, GC B cells and FDCs (**Figure E**) as well as the higher relative expression of CXCR3 in mediastinal lymph nodes compared to cervical and mesenteric lymph nodes indicated an active immune response to viral infection (Fig. 3F). Consistently, we observed SARS-specific responses by GC T_fh_ cells in the mediastinal lymph node (Fig. 3G, Figure S4E).

### Humoral responses to SARS-CoV-2 are dominated by IgG antibodies

**Figure 4:**
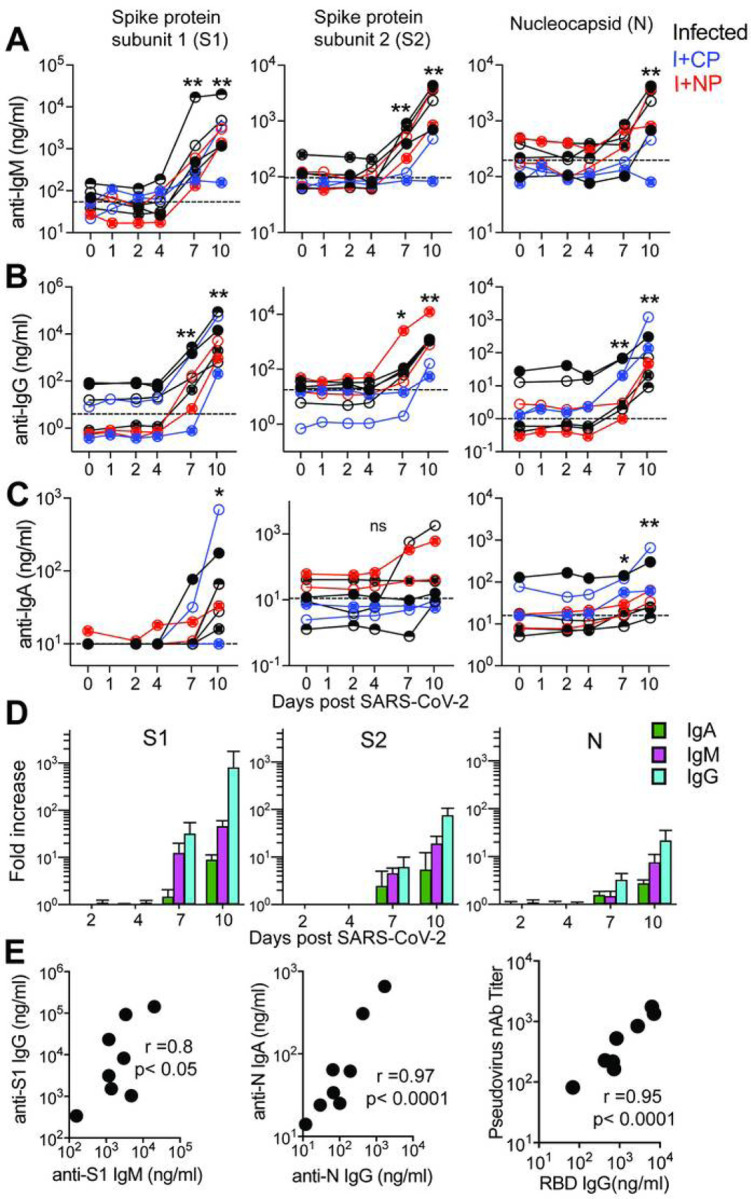
Humoral responses to SARS-CoV-2 are dominated by IgG antibodies Concentrations of (A) IgM, (B) IgG, and (C) IgA antibodies specific for S1, S2, and N proteins measured by BAMA or ELISA in serum. The dashed line represents the median pre-infection (day 0) concentration for all animals. (D) Fold increase in antibody responses in animals not given CP was determined by dividing post-infection concentrations by those measured on day 0 in each animal. Geometric mean fold increases with SEM are shown. (E) Correlations between day 10 levels of S1 -specific IgG and IgM, N-specific IgA and IgG, and pseudovirus neutralizing antibody titers and anti-RBD IgG antibodies measured by ELISA. Unique symbols identify animals in each of the experimental groups.

Studies in humans demonstrate that greater than 90% of SARS-CoV-2 patients develop binding antibodies to S antigen within 10 days of symptom onset^26,27^. However, the kinetics of the early antibody response to S and N proteins and the contributing antibody isotypes, specifically in the setting of mild or asymptomatic clinical illness, are not well-defined. Here, we quantified concentrations of serum antibodies to S1, S2, and N antigens, and used a secondary antibody specific for macaque IgG to distinguish *de novo* IgG antibodies from passively infused human CP IgG antibodies. The data showed IgM and IgG seroconversion to S1 and S2 proteins in all animals by day 7 post-infection, with the exception of one animal (Fig. 4A & B). This is consistent with reports that S- or RBD-specific IgG and IgM antibodies often appear simultaneously in blood of most humans infected with 2002 SARS or CoV-2^26–28^. Antibody responses to the N protein in humans are reported to increase 10 days following disease onset^29,30^, and interestingly, N-specific IgG was evident in all macaques by day 7 but N-specific IgM was not increased significantly (3-fold over baseline values) until day 10 in most animals. In addition, 50% of the animals failed to demonstrate a significant IgA response to all SARS CoV-2 proteins within 10 days of infection (Fig. 4C). However, we should note that analysis of some necropsy sera suggested that IgA antibodies continue to increase after day 10 (data not shown).

Evaluation of the magnitude of post-infection antibody responses in animals that did not receive CP plasma clearly indicated that IgG dominated the humoral response to all SARS CoV-2 proteins (Fig. 4D). On day 10, we observed strong correlations between S1-specific IgG and IgM and between N-specific IgA and IgG (Fig. 4E). Also consistent with reports in infected humans, we observed a strong correlation between neutralization antibody titers and concentrations of anti-RBD IgG antibodies on day 10 (Fig. 4E). Together, these data show rapid development of binding and neutralizing antibodies following SARS-CoV-2 infection in the context of mild or absent clinical symptoms. The appearance of antiviral IgG antibodies by day 7 with delayed induction of IgA responses suggests that early class-switching occurs after SARSCoV-2 infection and is likely promoted by T_h_1-type T_fh_ cells.

## Discussion

The importance of CD4 T_fh_ cells in the generation of plasma cells, critical for persistent antibody, places a premium on understanding the T_fh_ and GC response following SARS-CoV-2 infection. The present study adds to our understanding of immune responses to SARS-CoV-2 in three significant ways. First, we demonstrate that robust T_h_1-T_fh_ responses are observed following SARS-CoV-2 infection. Second, T_fh_ responses focused on S and N are seen within lymph nodes, circulate through peripheral blood, potentially seeding the spleen. Lastly, we show that acute antibody kinetics are characterized by induction of IgG, predominantly to S1, indicative of early class switching. Taken together, these data demonstrate that productive T_fh_ responses are elicited following SARS-CoV-2 infection in healthy adult rhesus macaques. While the T_h_1-bias of T_fh_ cells following infection is expected due to the robust interferon response following SARS-CoV-2, this skewing is important to note, as weak interferon responses observed in COVID patients could hamper shoring up effective antiviral antibody and CD8 T cell responses^31^.

Several studies have examined the kinetics of antibody responses in humans after the onset of symptoms and three unifying themes emerge from these data. First, in the majority of patients, antibodies to RBD of the S1 subunit are induced between 8–10 days of symptom onset, and levels of these antibodies correlate strongly with neutralizing titers^26,27,32^. Second, plasma from the majority of COVID-19 convalescent patients does not contain high levels of neutralizing activity^33^. Third, plasma antibodies in infected individuals that do develop neutralizing antibodies are minimally mutated^34^.

These data suggest that CD8 T cells may contribute to the control of SARS-CoV-2, and while a protracted germinal center response may not be critical for the generation of neutralizing antibodies it could improve antibody durability by enhancing plasma cell numbers. Our data add to this developing narrative by showing that in the setting of mild/asymptomatic illness, antibody responses are generated and characterized by the predominance of IgG. Intriguingly, we observed that IgM and IgG antibodies to the S1, S2 and N proteins were produced concurrently.

While it remains unknown whether immune responses elicited when naturally infected by SARS-CoV-2 will protect from re-infection, studies in rhesus macaques show that infection does protect against rechallenge, 28–35 days post first infection, signifying that some degree of a protective immune response follows infection^21^. In this context, the finding that infection induces CD4 helper responses targeting major structural proteins on the virus suggests that infection is capable of producing effective CD4 help for CD8 T cells and antibody responses. Indeed, antibodies against the RBD region in S1 are elicited in the vast majority of COVID-19 patients along with robust CD4 T cell responses^27^. Our data show that spike epitopes are immunogenic to both T and B cells and suggests that induction of these responses by vaccines may confer protection. While our discovery of N- and S-specific CD4 T cells in the spleen is intriguing at present we are unable to distinguish whether these cells represent cells that are seeded from circulation or are elicited *de novo* via trafficked antigen. Further studies are needed to tease apart the possibilities as this is central to understanding determinants of protective immunity. In sum, the data suggest that vaccine platforms inducing T_h_1 CD4 helper and T_fh_ helper responses are likely to succeed in eliciting robust CD8 T cell and antibody responses against SARS-CoV-2. Similar observations of circulating T_fh_ cells in vaccinated and infected humans support this hypothesis^14,35,36^.

In summary, the current study adds to our understanding of the CD4 helper responses to SARS-CoV-2 infection and provides an important foundation for harnessing the mechanisms that stimulate robust CD4 T_fh_ responses in the context of an effective vaccine.

## Methods

### 

#### Rhesus Macaques.

Eight colony-bred Indian origin rhesus macaques *(Macaca mulatta)* were housed at the California National Primate Research Center and maintained in accordance with American Association for Accreditation of Laboratory Animal Care guidelines and Animal Welfare Act/Guide. As described by us^37^, strict social distancing measures were implemented at the CNPRC at the start of the pandemic in March to reduce risk of human-to-rhesus SARS-CoV-2 transmission. Animals were screened for SARS-CoV-2 and housed in barrier rooms with increased PPE requirements prior to study assignment. Animals were four to five years of age with a median weight of 8.6 kg (range 5.4–10.7 kg), were SIV- STLVSRV-. Animals were seronegative for SARS-CoV-2 at study initiation. Sex distribution within experimental groups was as follows; Infected (n=3 females, n=1 male); Infected + Convalescent Plasma (n=2 males); Infected + Normal Plasma (n=2 males). Table S1 provides details of the animals in the study. For blood collection, animals were anesthetized with 10 mg/kg ketamine hydrochloride injected i.m. For virus inoculation and nasal secretion sample collection, animals were additionally anesthetized with 15–30 ug/kg dexmedetomidine HCl inject i.m. and anesthesia was reversed with 0.07–0.15 mg/kg atipamezole HCl injected i.m.

#### Virus and inoculations.

SARS-CoV-2 virus was isolated from the nasal swab of a COVID-19 patient with acute respiratory distress syndrome admitted to University of California, Davis Medical Center, Sacramento^38^. Vero cells (ATCC CCL-81) were used for viral isolation and stock expansion. The passage 2 viral stock (SARS-CoV-2/human/USA/CA-CZB-59X002/2020) used for animal inoculations had a titer of 1.2×10^6^ PFU/ml (corresponding to 2×10^9^ vRNA) (Genbank accession number: MT394528). To recapitulate relevant transmission routes of SARS-CoV-2, animals were inoculated with 1 ml stock instilled into the trachea, 1 ml dripped intranasally, and a drop of virus stock in each conjunctiva.

#### Convalescent plasma and infusions.

Convalescent plasma was sourced from Vitalant and represented a pool from up to four donors. Plasma was pooled prior to infusion into monkeys. Pooled plasma had a nAb titer of 1:1149, binding antibody titers for SARS-CoV-2 antigen were as follows; anti-S1-IgG, 24.5 μg/ml; anti-S2 IgG, 2.9 μg/ml; anti-NC IgG, 10.7 μg/ml. Normal plasma was collected prior to the COVID-19 pandemic and was negative for SARS-Cov2 antibody. Concentrations were as follows; anti-S1-IgG, 0.00004μg/ml; anti-S2 IgG, 0.003 μg/ml; anti-N IgG, 0.001 μg/ml. Twenty fours following virus inoculation, animals were infused with plasma at 4 ml/kg volume (total volume infused was 33–39 ml) at an infusion rate of 1 ml/minute. Control animals (n=4) were not infused.

#### Specimen collection and processing.

On days 2, 4, 5, 7, 8, and 10, a 5-french feeding tube was inserted into the nasal cavity. 2 ml PBS were instilled through each nostril and the maximum volume was aspirated, secretions were spun at 800 g for 10 min and 1 ml of supernatant and cell pellet were lysed in 3 ml Trizol LS for RNA isolation. EDTA-anticoagulated blood was collected on Days 0, 2, 4, 7, and 10 for immunophenotyping. PBMCs were isolated from whole blood collected in CPT vacutainer tubes, sampled at Day 7 and necropsy, by density gradient centrifugation as previously described^39^. For serum, coagulated blood was centrifuged at 800 g for 10 min to pellet clotted cells, followed by extraction of supernatant and storage at −80°C. Lymph nodes, spleen, and lung tissue were obtained at necropsy and digested enzymatically using collagenase followed by manual disruption to obtain single cell suspensions for flow cytometry based assays.

#### Activation induced Marker (AIM) assay.

Cells were stimulated with overlapping peptide pools representing SARS-CoV-2 and responding cells were identified by upregulation of activation markers, as described previously^39,40^. All antigens were used at a final concentration of 2 mg/mL in a stimulation cocktail made with using 0.2 mg of CD28 and 0.2 mg CD49d costimulatory antibodies per test. Unstimulated controls were treated with volume-controlled DMSO (Sigma-Aldrich). Tubes were incubated in 5% CO_2_ at 37°C overnight. Following an 18 h stimulation, the cells were stained, fixed, and acquired the same day. AIM assays on splenocytes and mediastinal lymph nodes were performed on cryopreserved cells (Table S2). AIM assay on day 7 PBMCs were performed on fresh cells. Phenotype panel on LNs and PBMCs was performed using standard flow cytometry assays.

### vRNA quantitation by quantitative real time polymerase chain reaction (qRT-PCR)

Trizol lysed nasal samples were processed using a modified Qiagen RNeasy Lipid Tissue Mini Kit protocol. Briefly, 5.6ul polyacryl carrier was added to trizol lysate, followed by 1/10 volume BCP and phase separated as described in Qiagen protocol. 8ul of eluted RNA was DNase treated with ezDNase per kit instructions and converted to cDNA with Superscript IV using random primers in a 20ul reaction and quantified in quadruplicate by qPCR on an Applied Biosystems QuantStudio 12K Flex Real-Time PCR System using Qiagen QuantiTect Probe PCR Mastermix with primers and probe that target the SARS-CoV-2 Nucleocapsid (forward 5’-GTTTGGTGGACCCTCAGATT-3’, reverse 5’-GGTGAACCAAGACGCAGTAT-3’, probe 5’-/5-FAM/TAACCAGAA/ZEN/TGGAGAACGCAGTGGG/3IABkFQ/−3’).

#### Serum cytokines.

Serum cytokines. Luminex® (NHP Cytokine Luminex Performance Pre-mixed kit, R&D, FCSTM21) was performed to evaluate cytokines in rhesus macaque sera. The assay was performed according to the manufacturer’s protocol. The beads for each sample, control, and standard curve point were interrogated in a Luminex® 200 dual laser instrument (Luminex, Austin, TX) which monitors the spectral properties of the beads and amount of associated phycoerythrin (PE) fluorescence for each sample. xPONENT® software was used to calculate the median fluorescent index and calculate the concentration for each cytokine in

#### Flow cytometry.

Cell staining was performed as previously described. Whole blood and single cell suspensions from the lung and lymph nodes were stained fresh and acquired the same day. Staining on spleen was performed on cryopreserved samples. Fluorescence was measured using a BD Biosciences FACSymphony™ with FACSDiva™ version 8.0.1 software. Compensation, gating and analysis were performed using FlowJo (Version 10). Reagents used for flow cytometry are listed in Table S2.

#### BAMA for IgG and IgM antibodies to S1, S2 and N proteins

A customized BAMA was developed to simultaneously measure antibodies to the following recombinant SARS CoV-2 proteins (all from SinoBiologicals, Wayne, PA): S1 (#40591-V08H), S2 extracellular domain (#40590-V08B) and nucleocapsid (N; #40588-V08B). Briefly, proteins were dialyzed in PBS and conjugated to Bioplex Pro carboxylated magnetic beads (BioRad, Hercules, CA) as previously described 39. Standard and serum samples treated with 1% TritonX-100 detergent were serially diluted in PBS containing 1% BSA, 0.05% azide, and 0.05% Tween-20 and mixed with beads overnight at 1100rpm and 4°C. The following day, the beads were washed and treated with biotinylated antibody followed by neutralite avidin-phycoerythrin (Southern Biotechnology Associates: SBA, Birmingham, AL) as described (Phillips 2017). A BioRad Bioplex 200 and BioManager software were used to measure fluorescent intensity and construct standard curves for interpolation of antibody concentrations in test samples.

The standard was pooled serum from macaques infected for 11–14 days with SARS CoV-2. The following humanized (IgG1) monoclonal antibodies were used to estimate concentrations of IgG, IgM, and IgA antibodies in the rhesus serum standard: anti-S1 RBD (Genscript #HC2001), anti-S2 (SinoBiologicals #40590-D001) and anti-NC (Genscript #HC2003). Human and rhesus IgG antibodies were both detected in these calibration assays using biotinylated affinity-purified goat anti-human IgG y chain polyclonal antibody (SBA #2048–08). In subsequent BAMA assays for SARS CoV-2-specific rhesus macaque IgG antibodies, biotinylated mouse anti-monkey IgG y chain monoclonal antibody (SBA cat#4700–08) was used as the secondary antibody. IgM antibodies were detected using biotinylated affinity-purified goat anti-human IgM μ chain polyclonal antibody (SBA#2020–08) which cross-reacts well with macaque IgM. Results obtained for IgM in the rhesus standard were multiplied by 3.3 to account for under-estimation by the monomeric IgG monoclonal antibody standard. Macaque IgA antibodies were detected with the antibodies described below. IgG, IgM or IgA antibodies had to be increased 3-fold over the day 0 value to be considered significant

### ELISA for SARS-specific IgA and antibodies to RBD

These assays were done using methods similar to those described^41^ and Immulon 4 microtiter plates (VWR, Radnor, PA) coated with 100ng per well of S1, S2, N or RBD protein (SinoBiological #40592-VNAH). For IgA assays, a pooled rhesus serum collected day 14 after infection with SARS CoV-2 was used as standard after depletion of IgG using GE Healthcare Protein G Sepharose (Sigma, St. Louis, MO) as described^41^. Test sera were also depleted of IgG to facilitate detection of low levels of IgA antibodies. Macaque IgA was detected using a mixture of biotinylated clone 10F12 (NHP Reagent Resource) and biotinylated clone 40.15.5 (Ward et al 1995) anti-rhesus IgA monoclonal antibodies, which do not cross-react with human IgA and, when combined, appear to recognize all allotypes of rhesus macaque IgA (Kozlowski, personal observation). For RBD IgG assays, the above pooled rhesus serum standard and secondary monoclonal antibody specific for macaque IgG were used.

#### Neutralizing assay

Pseudovirus neutralization assay was performed as described^42^.

#### Statistics

Statistical analyses were performed using GraphPad Prism 8.4.2. Within group comparisons, such as immune responses and antibody levels at different time points, were done using the two-tailed Wilcoxon matched-pairs signed rank test. For correlation analysis, the two-tailed Spearman rank correlation test was used.

#### Materials Availability.

The study generated SARS-CoV-2/human/USA/CA-CZB-59X002/2020 and is available upon request from CJM (Genbank accession number: MT394528).

## Supplementary Material

Supplement

Supplement

Supplement

Supplement

Supplement

Supplement

Supplement

Supplement
