# Supplementary figures and images for: SARS-CoV-2 infection induces robust germinal center CD4 T follicular helper cell responses in rhesus macaques
Source: Res Sq. 2020 Aug 14:rs.3.rs-51545. Preprint. [Version 1] doi: 10.21203/rs.3.rs-51545/v1 (PMC7430596; doi:10.21203/rs.3.rs-51545/v1)

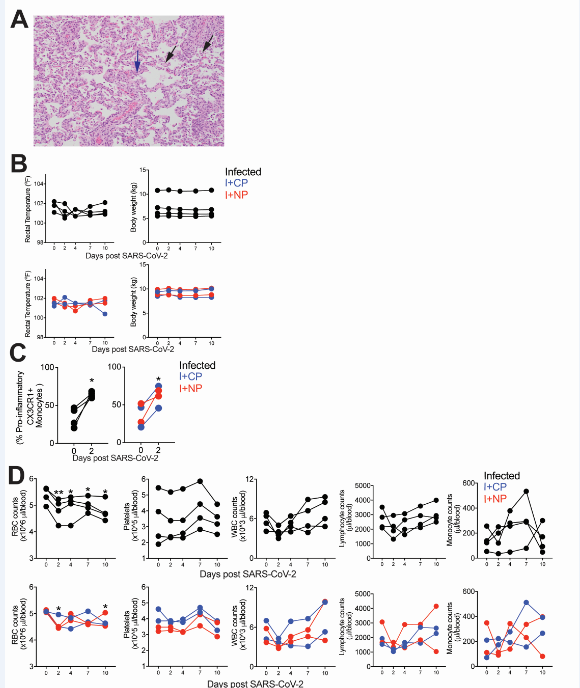

Supplement: Supplement [file FigureS1.png]

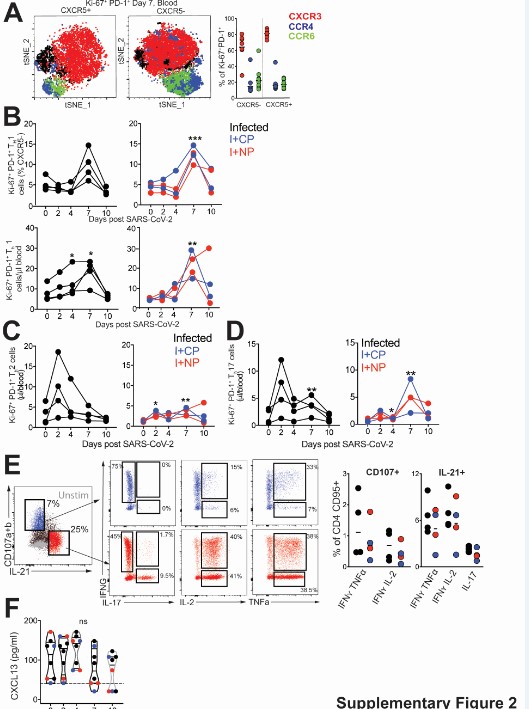

Supplement: Supplement [file FigureS2.jpg]

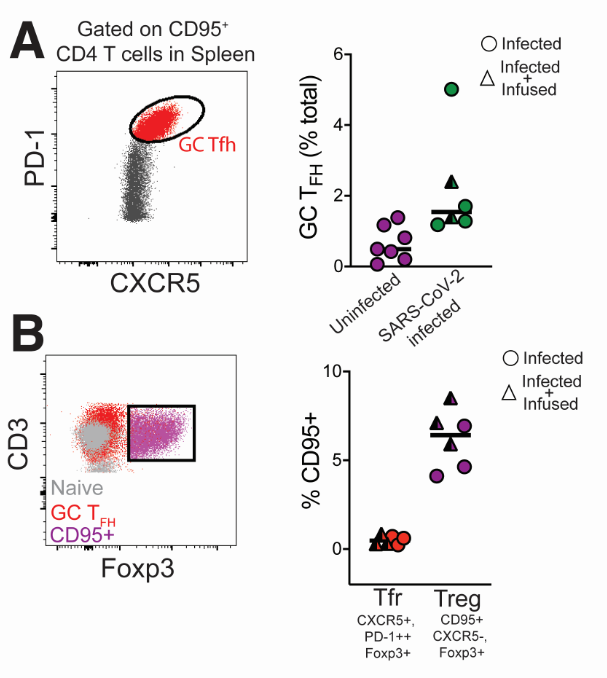

Supplement: Supplement [file FigureS3.png]

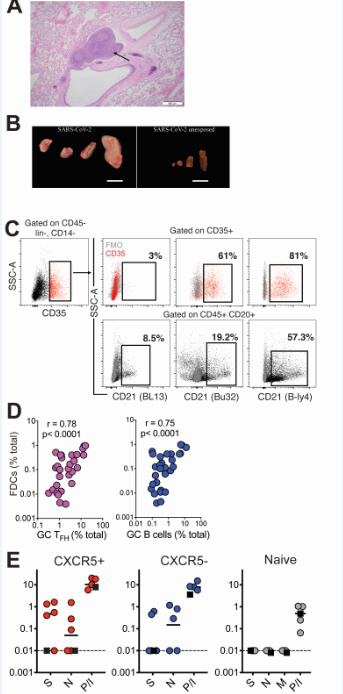

Supplement: Supplement [file FigureS4.png]

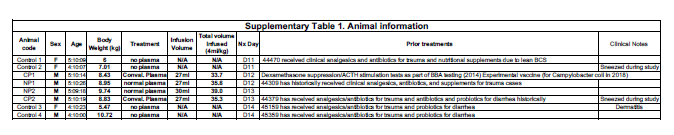

Supplement: Supplement [file TableS1.png]

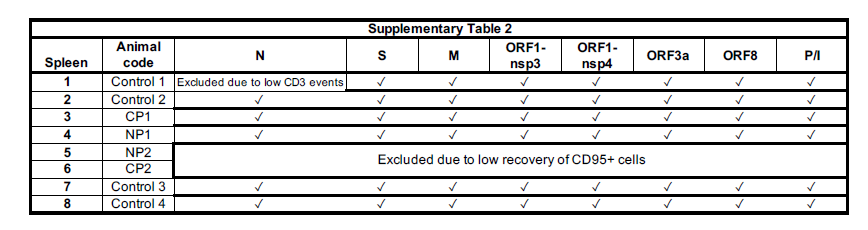

Supplement: Supplement [file TableS2.png]

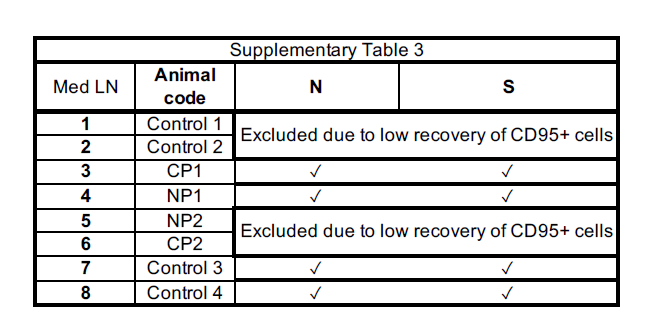

Supplement: Supplement [file TableS3.png]
